# Studies on the sequential pathology of Kyasanur Forest Disease (KFD) in Mouse brain: KFD virus induces apoptosis of neurons in cerebrum and hippocampus

**DOI:** 10.1371/journal.pone.0297143

**Published:** 2024-03-01

**Authors:** Ullasgowda K. Srikanth, Chandranaik B. Marinaik, Suguna Rao, Amitha Reena Gomes, Doddamane Rathnamma, Shrikrishna Isloor, Bharath T. Lakshmikanth, Chinmayie K. Siddaramegowda, Apsana Rizwan, Sonnahallipura M. Byregowda, Mudalagiri D. Venkatesha, Archana Munivenkatarayappa, Raveendra Hegde

**Affiliations:** 1 Institute of Animal Health and Veterinary Biologicals, KVAFSU, Bangalore, India; 2 Veterinary College, KVAFSU, Bangalore, India; University of Ibadan Faculty of Veterinary Medicine, NIGERIA

## Abstract

The sequential pathology of Kyasanur forest disease (KFD) in mouse brain was assessed in this study. Kyasanur forest disease virus (KFDV) strain P9605 used in this study was confirmed by real-time reverse transcriptase-polymerase chain reaction targeting the NS5 gene. Mouse Lethal Dose 50 (MLD_50_) of the virus was determined by *in-vivo* mice inoculation test. One MLD_50_ of the KFDV was inoculated intra-cerebrally into 36 mice aged 2–3 weeks. Another group of 36 age-matched mice that served as control group were inoculated with plain media. Six mice each from infected and control groups were euthanized every 24 hrs intervals for six days. Brain tissues were collected in 10% NBF. The collected brain tissues were processed and subjected to histopathological studies by Hematoxylin and Eosin staining. Grossly, the infected mice showed symptoms of dullness, hunched back appearance, weakness, sluggish movements with indication of hind quarter paralysis on day four post-infection. These symptoms got aggravated with complete paralysis of the hind quarters, inability to move and death on 5^th^ and 6^th^ day post-infection. Microscopically, the brain showed apoptosis of neurons, perivascular cuffing, gliosis, congestion, neuropil vacuolation, meningitis, degeneration, and necrotic neurons. The real-time RT-PCR on hippocampus of the KFDV-infected mouse brain showed three-fold higher expression levels of Caspase 3, a crucial mediator of apoptosis. The cerebral cortex, cerebellum and hippocampus that control the motor neuron activities and muscle tone were primarily affected, possibly correlating with the gross symptoms of hind quarter paralysis, ataxia, and other motor neuron dysfunctions noticed. Taken together, these findings reveal that KFDV induces apoptosis of neurons in the cerebrum and hippocampus of KFDV infected mice. Further studies are needed to confirm if the lesions noticed in mice brain simulate the brain lesions in humans since gross motor-neuron symptoms are similar in mice as well as humans.

## Introduction

Kyasanur forest disease (KFD) is an emerging vector-borne viral zoonosis. The disease is caused by Kyasanur forest disease virus (KFDV) belonging to the virus family *Flaviviridae* and the genus Flavivirus [1].

Kyasanur forest disease virus was discovered in 1957 during an outbreak in monkeys and humans at Kyasanur forest of Karnataka state in India [2]. The disease is transmitted to humans and monkeys mainly by the bite of KFDV-infected ticks of the *Haemaphysalis* species [3]. The virus is maintained in these forest environments by cycling with reservoir species such as rats, shrews, bats [4], and probably cattle [5, 6].

Humans may contract the disease by handling and interactions with diseased or dead monkeys when they reach forest areas to establish rice crops, pasture cattle, and to collect firewood [2]. During these activities, people come in close contact with infected ticks and possibly diseased or dead monkeys such as red-faced bonnets (*Macaca radiata*) and black-faced langur (*Semnopithecus entellus*) in forests [7]. Furthermore, climatic changes, increased deforestation, and agricultural activities have resulted in additional contacts between ticks, monkeys, and humans in KFD-endemic areas [8].

Kyasanur forest disease causes significant morbidity and mortality in humans. The burden of the disease is increasing with each passing year. The disease has re-emerged with known seasonal outbreaks and the virus has become a matter of concern due to its multiple re-emergence within a short period of time. There were 3075 positive KFD cases from 2000 to 2019, with 52 deaths in the state of Karnataka, India [9].

The disease has an incubation period of three to eight days and the illness begins with flu-like symptoms such as fever or headache, myalgia of back and extremities, diarrhea, vomition, conjunctivitis, prostration, and hypotension. More than half (54%) of the affected individuals will progress to hemorrhagic symptoms with palate eruptions, nose bleeding, bloody vomitus, hematuria, and dysentery. Many patients will have convulsions, neck stiffness, sensorium changes and other neurological symptoms. In later stages, patients are often quite weak and bed-ridden with muscle tremors, twitching, or trembling. The KFD has a case fatality rate of 3–5% and the deaths are related with complications due to bleeding, encephalitis, bronchopneumonia (secondary bacterial infections), shock (blood congealing or clotting inside the blood vessels), multi-organ/system failure, and renal or hepatic failure [10, 11].

Vaccination of susceptible population in and around the endemic area is the only way for effective control of the disease. The Institute of Animal Health and Veterinary Biologicals (IAH & VB) Bengaluru, produces a cell culture-based inactivated KFD vaccine for prophylactic use in people living in KFD-endemic regions, for the past 23 years. The IAH & VB is the only institute that produces vaccine against KFD in India. The vaccine and vaccination programmes have significantly helped people and has bought down the disease intensity, morbidity, sufferings, and mortality in the past 50 years [12]. As the course of disease can vary from subclinical infection to fatal cases with hemorrhagic and/or neurological complications, the pathology of KFD remains incompletely understood. Hence, the current work was taken up with an objective to study the sequential experimental pathology of Kyasanur forest disease in mouse brain and to draw possible correlation of the histopathology induced by the virus with the gross pathological changes observed.

## Materials and methods

### Confirmation of Kyasanur forest disease virus by real-time reverse transcriptase-polymerase chain reaction

The Kyasanur forest disease seed virus strain P9605 provided by National Institute of Virology, Pune, India was used in this study to evaluate the sequential pathology of KFD in mouse brain. The seed virus was confirmed by real-time reverse transcriptase-polymerase chain reaction (Real time RT-PCR). The RNA was extracted from the mouse brain suspension containing the KFD seed virus as per the procedure described in the QIAamp® Viral RNA Extraction Kit (Qiagen). The extracted RNA was subjected to Real-time RT-PCR as per the procedure described by Mourya and co-workers [13] in a QIAGEN Rotor-Gene Q thermal cycler. The primers and the probe used for the study were: Forward primer 5’ TGGAAGCCTGGCTGAAAGAG 3’, Reverse primer 5’ TCATCCCCACTGACCAGCAT 3’ and the TaqMan probe 5’ ATGGAGAGGAGCGCCTGACCCG 3’. The PCR was run with one cycle of reverse transcription at 50°C for 30 min and Taq inhibitor inactivation at 95°C for 10 min followed by 40 repeated cycles of 95°C for 15 Sec and 60°C for 1 min, amplifying a specific fragment of 63 bp amplicon of NS5 gene of KFDV.

### Sequential experimental pathology of Kyasanur Forest Disease virus in mouse brain

Mouse lethal dose (MLD) causing death in 50% of the mice inoculated with KFD seed virus (1MLD_50_) was used to study the sequential pathology in mice by intra-cerebral route of inoculation.

#### Determination of MLD_50_ of the KFD virus

The MLD_50_ of the KFD seed virus required for this study was determined as per the procedures of Ullas and co-workers [12], Rovozzo and Burke [14] and Dandawate [15]. The brief procedure is as follows:

Serial ten-fold dilutions of the KFD seed virus P9605 was done in glass vials with 0.50 ml of virus in 4.50 ml maintenance medium from 10^−1^ to 10^−8^. Five mice of 3–4 weeks old were injected intra-cerebrally with 0.03 ml of each of the dilutions starting from 10^−1^ to 10^−8^ (five mice in each dilution). Plain MEM was injected intra-cerebrally to five mice that served as control group. Another group of five mice were inoculated intra-cerebrally with undiluted KFD seed virus that served as positive control. All mice were observed daily for 14 days for disease symptoms at every 12 hour intervals. The KFD symptoms of hind quarter paralysis and inability to move in mice were taken as endpoint symptoms for computing the survival graphs. Mice showing symptoms of hind quarter paralysis and inability to move were humanely euthanized. Titre of the virus was calculated by the Reed and Muench method [16] and was expressed as MLD_50_/0.03 ml.

#### Procedure for mice inoculation and collection of brain sample

For studying sequential pathology, 0.03 ml of plain MEM containing 1MLD_50_ (10^6.3^/0.03 ml) of KFD virus was inoculated to 36 mice (3–4-week-old) by intra-cerebral route. Another group of 36 age-matched mice was injected with 0.03 ml of plain MEM by intra-cerebral route that served as control group. Each of six mice from infected and control group were euthanized at 24 hours intervals, for six days. The mice were euthanized using overdose of Isoflurane anesthetic. Under laminar air flow, the skulls of the mice were opened without damaging the brain. The brain tissues were carefully scooped/excavated out using spatula, without causing damage to the brain structures. Brain tissues were collected in 10% NBF and subjected to histopathological studies.

#### Procedure of sectioning and staining of brain tissue

The brain tissues were prepared for histopathological examination and stained with Hematoxylin and Eosin (H & E) as per the procedure outlined by Luna in 1968 [17]. For sectioning, the brain tissue was fixed in 10% NBF solution and washed with water to remove formalin. The tissue was then immersed in ascending grades of alcohol, beginning from 50% to absolute alcohol for an hour each, for the purpose of dehydration, then cleared in two changes of xylene for an hour each. The brain tissue was immersed in heated paraffin wax for impregnation for 3 hrs and then embedded in paraffin wax blocks and allowed to solidify. Using microtome, 3–5 μm thick sections were cut and slides were prepared. The sections were then stained with Hematoxylin and Eosin as per the procedure outlined by Luna [17] followed by mounting with DPX. The slides were examined under microscope and observations were recorded.

### Confirmation of apoptosis in mouse brain by estimation of Caspase 3 levels in hippocampus of mouse brain

To further confirm apoptosis, we employed real time RT-PCR to quantify the expression levels of caspase-3, a crucial mediator of programmed cell death [18]. For this, we inoculated 1MLD_50_ of KFD virus intra-cerebrally to six mice (3–4 weeks old), another set of six age-matched mice were inoculated with plain medium to serve as control group. Mice in both groups were euthanized on the sixth day post infection (dpi) and brains were collected as previously described.

The RNA was extracted from the mouse brain hippocampus as per procedure described in QIAamp® Viral RNA Extraction Kit procured from M/s Qiagen. The cDNA was synthesized using reverse transcription with a system of 20 μl comprising of 4 μl of 5x reaction mix, 1 μl of reverse transcriptase, 8 μl of extracted RNA and 7 μl of nuclease free water. The synthesized cDNA was subjected to Real-time PCR in a QIAGEN Rotor-Gene Q thermal cycler by SYBR Green method. The primers used for amplification of Caspase-3 in the study included; Forward primer 5’-GTGGAACTGACGATGATATGGC-3’, Reverse primer 5’-CGCAAAGTGACTGGATGAACC-3.

The β-actin was used as an endogenous control and the primers used for β-actin amplification included; Forward primer 5’-TGTGGATGACTGACTACCTGAACC-3’, Reverse primer 5’-CAGCCAGGAGAAATCAAACAGAGG-3’. Real-time PCR was carried out in a reaction volume of 25 μl with reaction conditions of: 95˚C for 30 sec, followed by 40 cycles of 95˚C for 5 sec, 60˚C for 30 sec and 72˚C for 60 seconds. Each sample was run twice. The relative expression was determined by the 2^−ΔΔCt^ method [19, 20].

ΔCt = Ct (gene of interest (Caspase 3) − Ct (Beta actin (housekeeping gene))

ΔCt is the value obtained by subtracting the Ct value of the internal gene from the gene under investigation for a given sample. The internal control gene is not affected by the experiment, and it is used to normalise the value of interested gene. The delta-delta Ct (ΔΔCt) values show the difference between the samples and the controls. The final results of the analysis are then indicated by 2^−ΔΔCt^ as a fold change of the gene expression. Statistical analysis was performed using GraphPad Prism software.

### Regulatory permission to conduct research on KFD

Regulatory permissions from Institutional Animal Ethics Committee (IAEC) to conduct research on KFD in mice at ‘KFD vaccine production laboratory’ of Institute of Animal Health and Veterinary Biologicals, Bengaluru, India was accorded vide order, IAEC.CODE: IAH/IAEC/BP/PE/KFD/2021-22/13, dated 26/08/2021.

Also, approval from Institutional Bio Safety Committee (IBSC) was accorded to conduct this research vide order IAH:IBSC:Project number-07-08/21, dated 26/08/2021.

## Results

### Confirmation of the Kyasanur Forest Disease (KFD) virus

**Fig 1:**
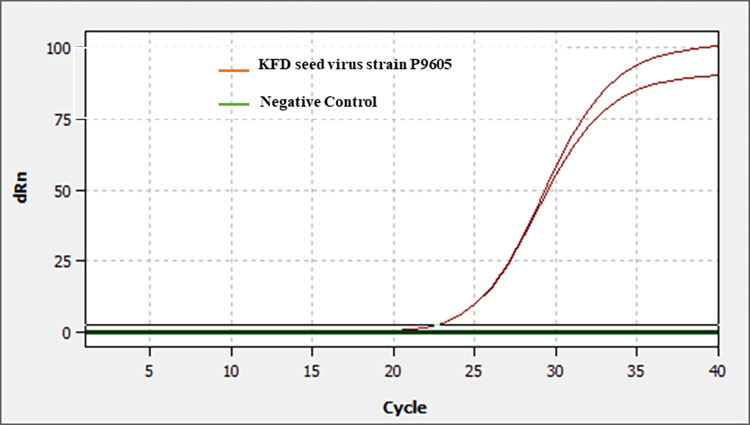
Real-time PCR confirmation of KFD seed virus. The RNA extracted from the mouse brain suspension containing the KFD seed virus was subjected to Real-time PCR as per the procedure described by Mourya and co-workers [13]. The PCR yielded specific amplifications indicating the presence of KFD virus in the seed stock of mouse brain suspension.

The seed virus was confirmed by real time polymerase chain reaction. The RNA extracted from the seed virus was subjected for real time PCR. The PCR yielded specific amplifications indicating the presence of KFD virus in the seed stock of mouse brain suspension (Fig 1).

### Determination of MLD_50_ of the KFDV by mice inoculation test

**Fig 2:**
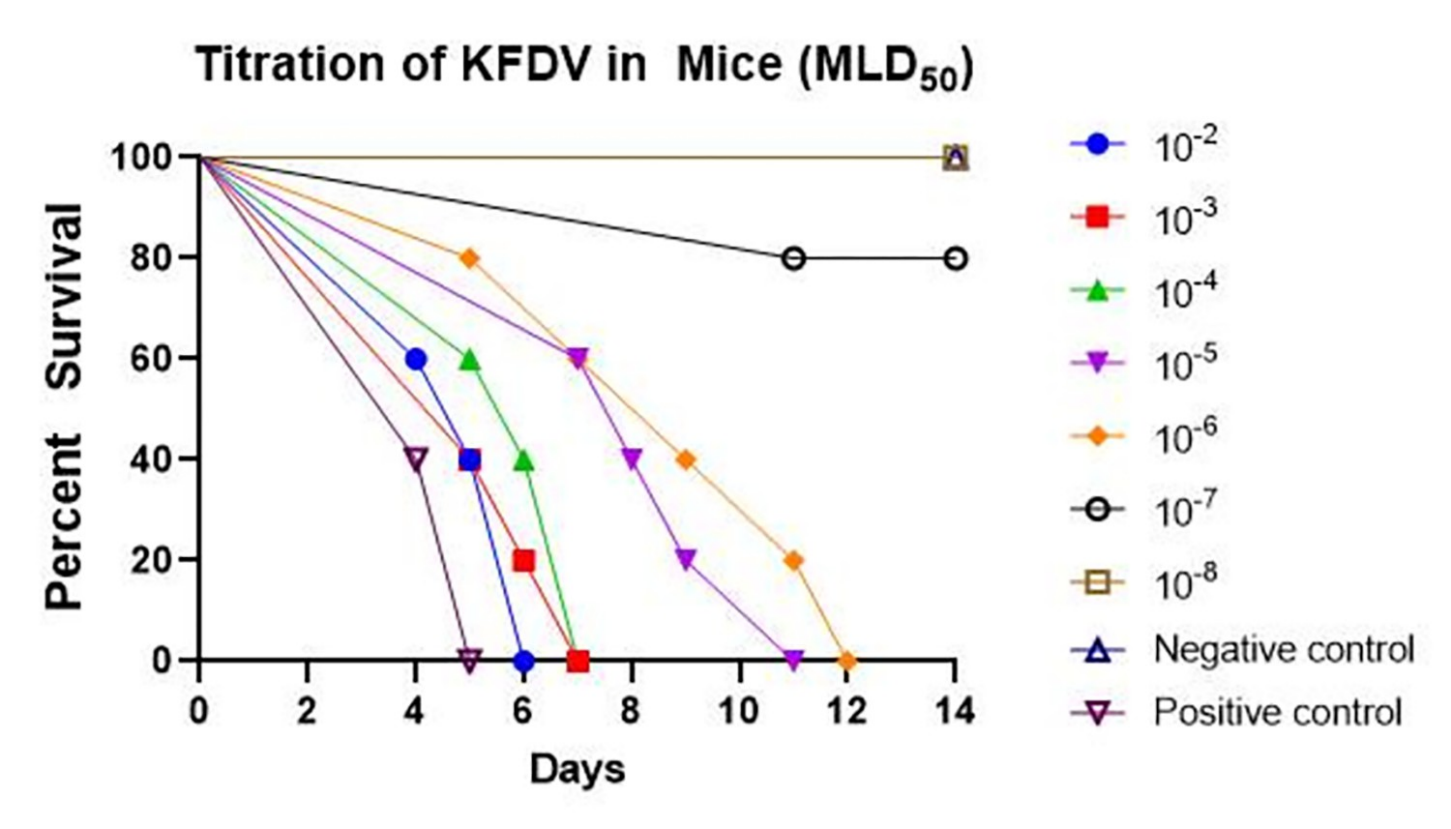
In vivo titration of KFDV (MLD_50_) by mice inoculation test. Each dilution of the KFD seed virus was injected intra-cerebrally to five mice of 3–4 weeks old. Plain medium was injected intra-cerebrally to a group of five mice that served as control group and another five mice were injected with undiluted seed virus that served as positive control. Mice were observed for 14 days at 12-hour intervals for disease symptoms. At the end of 14 days, virus dilutions of 10^⁻2^, 10^⁻3^, 10^⁻4^, 10^⁻5^ and 10^⁻6^ caused disease symptoms in 100% of mice, whereas virus dilution of 10^⁻7^ caused disease in 20% of mice and virus dilution of 10^⁻8^ did not cause symptoms of the disease in any mice. The control group of mice that received plain medium remained healthy during the study period. All the mice that received undiluted virus showed classical symptoms of KFD.

Each dilution of the KFD seed virus from 10^−1^ to 10^−8^ dilutions was injected intra-cerebrally to five mice of 3–4 weeks old. Plain medium was injected intra-cerebrally to a group of five mice that served as control group and another five mice were injected with undiluted seed virus that served as positive control. Mice were observed for 14 days at 12-hour intervals for disease symptoms (Fig 2).

At the end of 14^th^ day, all the mice that received virus dilutions of 10^⁻2^, 10^⁻3^, 10^⁻4^, 10^⁻5^ and 10^⁻6^ showed disease symptoms / sickness. One mouse in the group that received virus dilution of 10^⁻7^ showed symptoms of hind quarter paralysis and all mice in the group that received virus dilution of 10^⁻8^ did not show disease symptoms (Fig 2). The control group of mice that received plain MEM remained healthy during the study period (Fig 2). All the mice that received undiluted virus showed classical symptoms of KFD.

Virus dilutions of 10^⁻2^, 10^⁻3^, 10^⁻4^, 10^⁻5^, 10^⁻6^ caused disease symptoms in 100% of mice, whereas virus dilution of 10^⁻7^ caused disease in 20% of mice and virus dilution of 10^⁻8^ did not cause symptoms of the disease in any mice. Thus, 1MLD_50_ of the KFD seed virus (Reed and Muench method) was 10^−6.375^/0.03 ml (Table 1).

**Table 1:** Determination of MLD_50_ of the KFD seed virus by i*n-vivo* titration in mice. Each dilution of the KFD seed virus was injected intra-cerebrally to five mice of 3–4 weeks old. Plain medium was injected intra-cerebrally to a group of five mice that served as control group and another five mice were injected with undiluted seed virus that served as positive control. Mice were observed for 14 days at every 12 hour intervals for disease symptoms. At the end of 14 days, virus dilutions of 10^⁻2^, 10^⁻3^, 10^⁻4^, 10^⁻5^, 10^⁻6^ caused disease symptoms in 100% of mice, whereas virus dilution of 10^⁻7^ caused disease in 20% of mice and virus dilution of 10^⁻8^ did not cause symptoms of the disease in any mice. Thus, the one MLD_50_ of the KFD seed virus (Reed and Muench method) was 10^−6.375^/0.03 ml.

Determination of MLD_50_ of KFD virus							
Virus Dilution	Mortality ratio	No. Infected	No. uninfected	Cumulative		Infection Ratio I ___ (I) + (UI)	Percent Mortality
				Infected (I)	Uninfected (UI)		
**10¯** ^**2**^	5/5	5	0	26	0	26/26	100%
**10¯** ^**3**^	5/5	5	0	21	0	21/21	100%
**10¯** ^**4**^	5/5	5	0	16	0	16/16	100%
**10¯** ^**5**^	5/5	5	0	11	0	11/11	100%
**10¯** ^**6**^	5/5	5	0	6	0	6/6	100%
**10¯** ^**7**^	1/5	1	4	1	4	1/5	20%
**10¯** ^**8**^	0/5	0	5	0	9	0/9	0%

Log MLD_50_ = Log Dilution above 50% mortality–(PD × Log Dilution factor)

= - 6 –(0.375 × 1) = - 6.375

1MLD_50_ = 10^−6.375^/0.03 ml

The Proportional Distance (PD) is the actual end point dilution (the dilution which would give an exact 50% mortality) in mice. PD was calculated by Reed and Muench formula:

$$Proportional\;Distance\;(PD)=\frac{Percent\;mortality\;next\;above\;50\%‐50}{Percent\;mortality\;next\;above\;50\%‐Percent\;mortality\;next\;below\;50\%}$$

### Sequential experimental pathology of Kyasanur Forest Disease in mouse brain

The following are the gross and histopathological changes observed after inoculating one MLD_50_ of KFDV intra-cerebrally in mice.

### Gross pathological findings in KFDV infected mice

**Fig 3:**
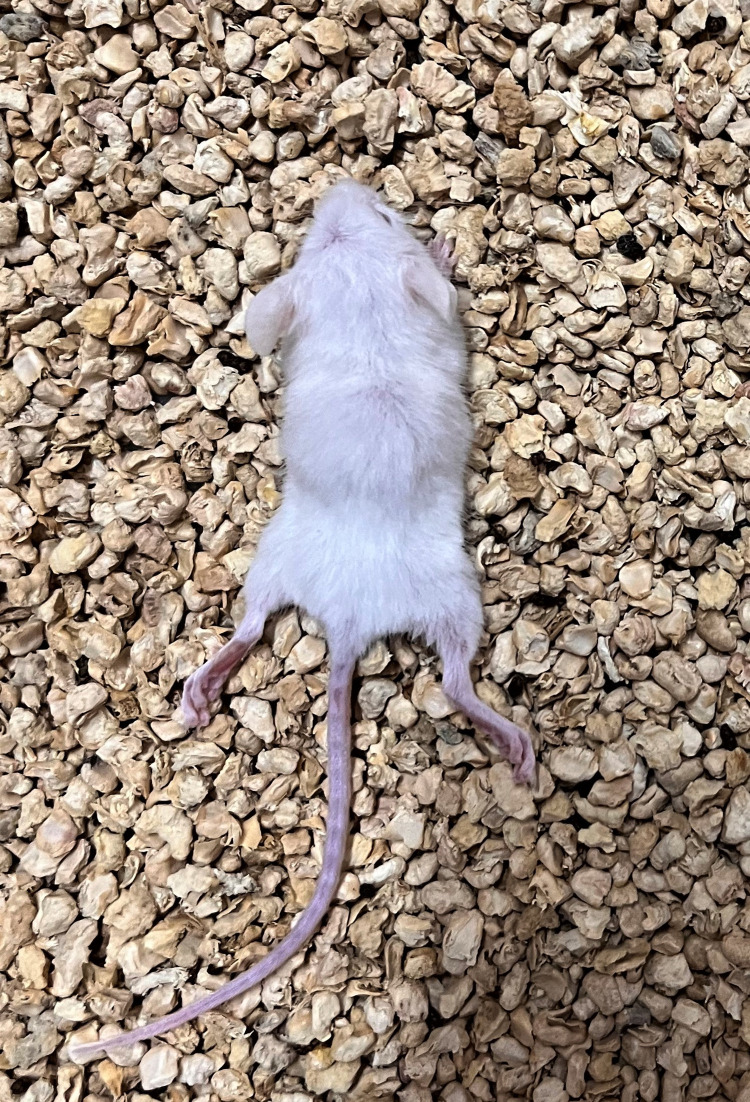
Hind quarter paralysis observed in KFDV-infected mice.

After infection with KFDV to mice, no symptoms were observed till 3^rd^ day post-infection (dpi). From the 4^th^ dpi, infected mice showed symptoms of ruffled fur, sleepiness, and hunched back. On the 5^th^ dpi, mice in the infected group showed slow gait, dullness, hunched back appearance, and hind quarter paralysis (Fig 3), and movement with dragging of the hind quarters. On day 6 post-infection, mice in the infected group showed complete paralysis of the hind quarters with increase in intensity of symptoms noticed on day 5 post-infection. The un-inoculated control mice and mice inoculated with plain MEM only did not show any disease symptoms throughout the study period. The brain tissues were carefully collected at 24-hour intervals in 10% NBF.

### Histopathological changes in mice brain infected with KFDV

**Fig 4:**
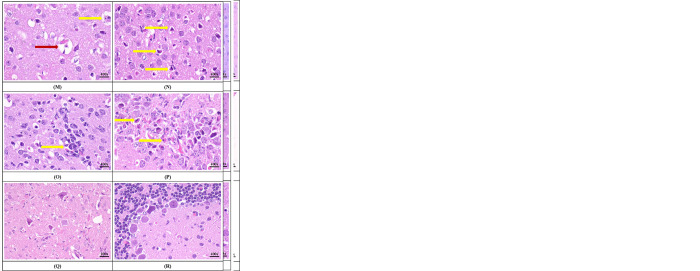
Sequential histopathological changes in mice brain following intra-cerebral infection with KFDV (Hematoxylin and Eosin staining as per the procedure of Luna [17]). A: Day 1- Traumatic hemorrhage in brain, post infection/injection, B: Day 1- Normal appearance of cerebrum, C: Day 1- Normal appearance of hippocampus, D: Day 3- Normal appearance of cerebrum with mild meningeal congestion, E: Day 4- Congestion of meningeal blood vessels, F: Day 5- Meningeal congestion and slow infiltration of inflammatory cells, G: Day 5- Congestion of blood vessels in the cerebrum, H: Day 5- Congestion and perivascular cuffing in the cerebrum, note almost normal appearing neurons in the adjacent areas, I: Day 5- Mild perivascular cuffing and occasional neuronal cell necrosis in the cerebrum, J: Day 6- Meningitis with congestion and infiltration of inflammatory cells, K: Day 6- Vascular congestion and perivascular cuffing in cerebrum, L: Day 6- Microglial aggregation around a vessel. Also note vacuolation of neuropil, degenerated and necrotic neurons in the adjacent area, M: Day 6- Vacuolization of neuropil and presence of necrotic (Red arrow) and apoptotic neurons (Yellow arrow), N: Day 6- A large number of apoptotic neurons (arrow) in the cerebrum, O: Day 6- Microglial aggregation, neuropil vacuolation, necrotic and apoptotic neurons in the cerebrum, P: Day 6- A large number of apoptotic neurons (arrow) in the pyramidal layer of the hippocampus, Q: Day 6- Shrunken neurons with condensed cytoplasm and pyknotic nucleus, in the mid-brain, R: Day 6- Condensation of occasional neuron in the Purkinje cell layer of the cerebellum.

On day 1, microscopic examination of the brain in KFDV-infected mice revealed multifocal area of post-traumatic hemorrhage (due to intra-cerebral injection) in the cerebral cortex (Fig 4A). The cerebrum (Fig 4B) and the hippocampus (Fig 4C) in the brain appeared normal.

The persistence of post-traumatic hemorrhage (due to intra-cerebral injection) in the cerebral cortex was observed on day 2. The other parts and cellular components of the brain remained apparently normal.

On day 3 following KFDV infection, hemorrhage due to traumatic injury (due to intra-cerebral injection) was reduced in severity in the cerebral cortex. Mild congestion of the meningeal blood vessels was observed (Fig 4D). However, the other parts and cellular components of the brain appeared normal.

On day 4, mild congestion was observed in the blood vessels involving meninges, cerebral cortex and cerebellum. Mild segmental meningeal hemorrhage was observed (Fig 4E). The cellular components including neurons appeared unaffected.

On day 5, progressive increase in the severity of congestion and hemorrhage was observed involving meninges along with mild infiltration of inflammatory cells indicative of meningitis. In addition, congestion of blood vessels was observed in the cerebrum, hippocampus and cerebellum with perivascular cuffing with less number of inflammatory cells. The neurons were degenerative and necrotic in the cerebrum and appeared shrunken with condensed nucleus and darkly stained cytoplasm (Fig 4F–4I).

On day 6, there was further increase in the severity of meningitis with severe congestion, hemorrhage, cellular infiltrations, and meningeal thickening. In cerebrum, a large number of blood vessels showed congestion and severe degree of perivascular cuffing with mononuclear cells (Fig 4J and 4K).

Neuropil vacuolation was observed in the cerebrum giving vacuolar appearance and large number of neurons were degenerative, necrotic, and apoptotic (Fig 4L). Apoptotic neurons appeared shrunken with empty space around and possessed with condensed or fragmented nucleus and dense eosinophilic cytoplasm [Fig 4M (Yellow arrow), Fig 4N (Yellow arrows)].

Degenerative and necrotic neurons appeared swollen containing vacuolated cytoplasm with dissolution of the nucleus [Fig 4M (Red arrow)]. In addition, small numbers of microglial aggregation was observed at multiple locations (Fig 4O). The pyramidal layer of hippocampus also revealed a large number of degenerative, necrotic, and apoptotic neurons (Fig 4P) along with microglial infiltration. Large neurons in the hind brain were also degenerative and necrotic having condensed nucleus and dense eosinophilic to amphophilic cytoplasm (Fig 4Q). In the cerebellum, very few Purkinje cells were affected which were condensed (Fig 4R).

### Histopathological changes in control mice

**Fig 5:**
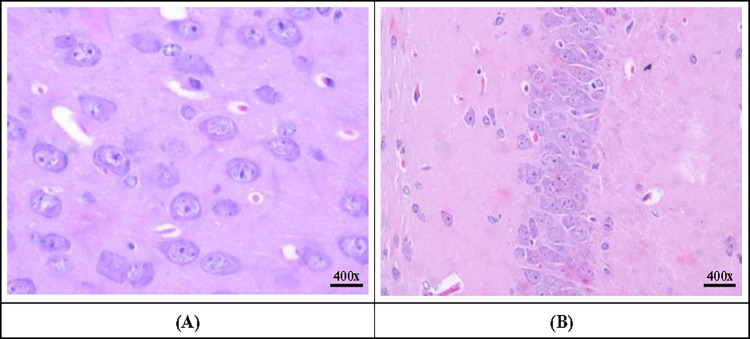
Sections of brain showing normal architecture of cerebrum and hippocampus in control mice (Hematoxylin and Eosin staining as per the procedure of Luna [17]). A: Normal appearance of neurons in the cerebrum, B: Normal appearance of neurons in the hippocampus.

On the first and second day after intra-cerebral inoculation of plain MEM into the control mice, multifocal areas of post-traumatic hemorrhage (due to intra-cerebral injection) in the cerebral cortex were observed, that reduced on day three and four post-inoculation. All the parts of the brain appeared normal with normal morphology and architecture including both parts of the cerebral cortex, seven layers of cerebrum (Fig 5A), pyramidal layer of hippocampus (Fig 5B) and Purkinje cells of cerebellum. The neurons appeared normal without any eosinophilic cytoplasm, degeneration or necrosis or karyorrhectic nucleus.

### Confirmation of apoptosis in mouse brain by estimation of Caspase-3 levels

**Fig 6:**
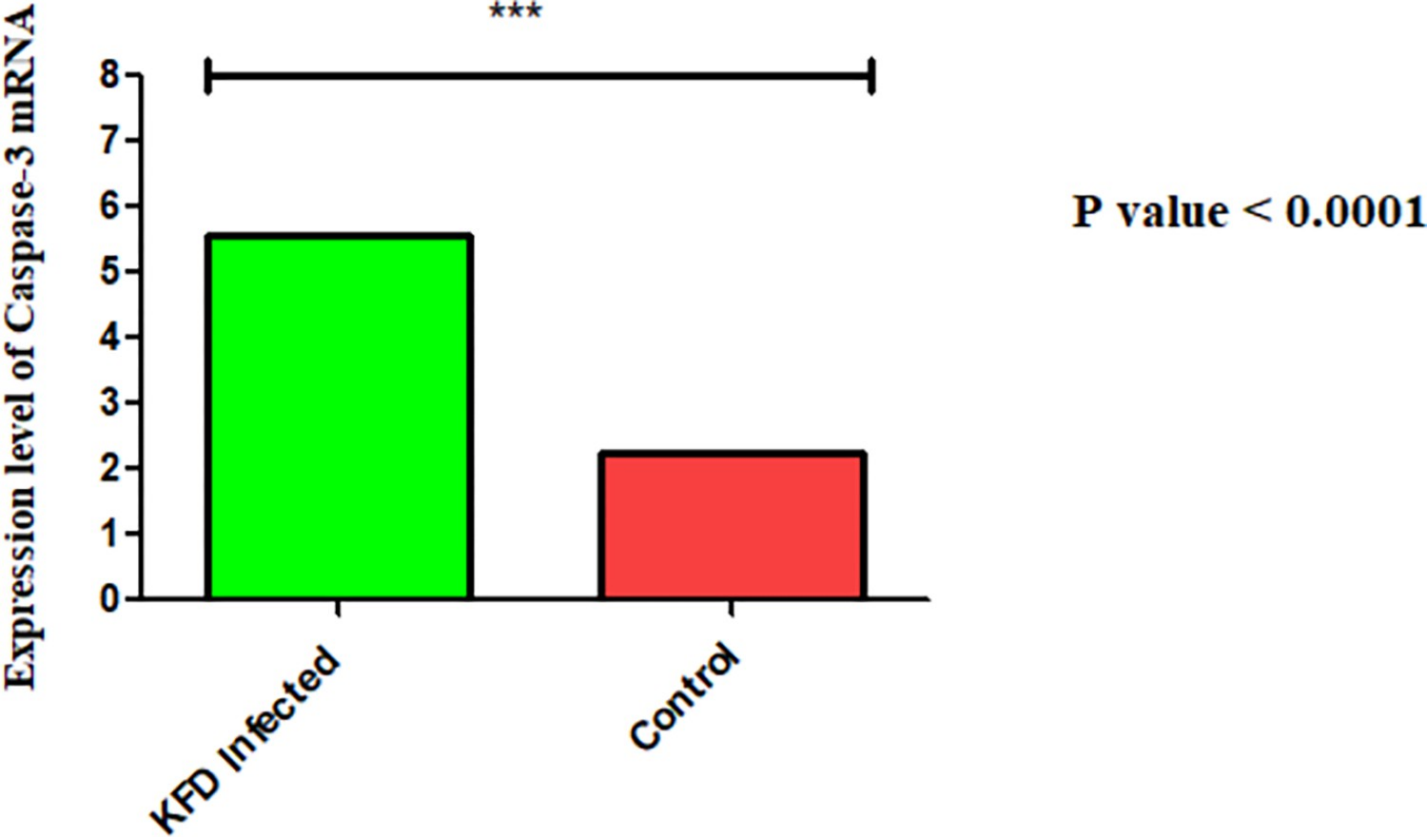
The expression levels of Caspase-3 mRNA in KFDV-infected and control mice brain. The real-time PCR on the RNA extracted from the hippocampus of the KFDV-infected mice brain and control mice brain showed a three-fold increase in Caspase-3 mRNA levels in virus-infected hippocampus compared to the control group (determined by the 2^−ΔΔCt^ method [19, 20]).

The real-time PCR on the RNA extracted from the hippocampus of KFDV-infected and control mice showed a three-fold increase in Caspase-3 mRNA levels in virus-infected hippocampus compared to the control mice (Fig 6).

## Discussion

Viral infections affecting the nervous system cause some of the most severe and feared diseases of humans; for example, rabies, herpes simplex encephalitis, poliomyelitis and arthropod-borne encephalitis. These diseases are often dramatic in their rapid onset and progression to coma or paralysis and death. In those who survive, permanent neurological deficits such as mental retardation, paralysis and parkinsonism are often the residua of the infection [21]. For each of these virus-induced diseases, neurons are the primary cells that are infected in the central nervous system. There is considerable evidence that both fatal disease and long-term sequelae in those who survive are caused by virus-induced death of neurons. However, remarkable recoveries can also occur. The process of cell death during virus infection is likely to be influenced by the types and maturity of the neurons infected, the virulence of the virus and the host immune response [21].

In humans, Kyasanur forest disease causes illness ranging from subclinical infection to severe disease with hemorrhagic/neurologic complications leading to death. The pathogenesis and pathology of KFDV have remained incompletely understood. Although the disease was initially thought to be hemorrhagic [22], later research has suggested that KFDV infections include a significant neurological component [23]. In this study, the sequential pathology of KFD in mouse brain was assessed, wherein the cellular changes in the brain were correlated with the gross pathology observed in KFDV-infected mice.

As stated previously, in this study the cerebrum and the hippocampus were highly affected, the cerebellum and mid-brain were moderately affected, and other parts of the brain were least affected. The previous histopathological studies conducted in KFDV-infected mice have described findings of meningoencephalitis with lymphocytic and histiocytic perivascular cuffing and acute neuronal necrosis in mice [24–27]. Additionally, the current study established apoptosis of neurons mainly in cerebrum and hippocampus as the most critical, consistent and characteristic histopathological finding in KFD-affected mice. Further, the real-time PCR showed significantly higher expression levels of caspase-3 (three-fold) in the hippocampus of KFDV-infected mouse brain. These findings possibly indicate that KFDV causes neuronal loss mainly through inducing apoptosis.

Previously well-established studies indicate that different sections of the brain control different functions of the body [28]. Accordingly, the cerebrum and hippocampus are the main regions of the brain that regulate motor neuron functions and co-ordinate movements; the cerebellum controls the motor activity and muscle tone. As observed in this study, the cerebrum, hippocampus, and cerebellum were the most affected brain areas and this possibly correlates with the gross symptoms of paralysis of hind quarter, ataxia, and other motor neuron dysfunctions noticed in virus-inoculated mice.

Viruses from several virus families can infect neurons in the brain and spinal cord, and these viruses often preferentially infect specific populations of neurons, giving rise to characteristic diseases; for example, poliovirus infects motor neurons, causing paralysis, and Japanese encephalitis virus often targets basal ganglia neurons, causing parkinsonian symptoms [29]. However, the underlying processes influencing neuronal tropism are not well characterized. In our study, we found that KFDV preferentially causes apoptosis of neurons in the cerebrum and hippocampus in mouse model that correlate with the gross motor neuron disturbances observed in KFD-affected mice.

Apoptosis is an intracellular process involving a cascade of events that are regulated by a wide variety of cellular factors that inhibit and facilitate the process of programmed cell death [30, 31]. Initiation of the apoptotic process often begins at the cell surface and typically involves trans-membrane signalling and activation of caspases, a family of cysteine proteases that cleave key cellular substrates that facilitate cell death. There are many checks and balances associated with initiation of the apoptotic cascade. Viruses and viral proteins can interact with these cellular processes at multiple levels. The specific caspases responsible for cleaving particular targets inside cells are largely unknown. Caspase-3, generally considered to be an executioner caspase, is responsible for many late-stage proteolytic cleavages. For instance, caspase-3 causes cellular apoptosis by activating the DNase that degrades chromosomal DNA or by cleaving anti-apoptotic protein Bcl2 [30, 31]. In our study, we found that the levels of caspase-3 in the hippocampus of KFD-affected mice were three times more than the levels in control mice. However, the mechanisms of caspase-3 activity in KFDV-infected mice need to be further explored.

Apart from caspase-mediated intrinsic cellular signalling pathways, cellular apoptosis may also be induced by external death domain receptor through Fas/CD95 recruiting FADD (Fas-associated protein with death domain) [30, 31]. It is evidently not clear what factors are responsible for determining the apoptotic pathway the virus and/or the host selects during a viral infection. Over the years, scientists have been attempting to decipher this inter-relationship between the host, virus and the process of apoptosis but to date, no definite conclusion has been drawn on this. It will not be surprising if viruses cause neuronal apoptosis by mechanisms that are yet to be explained.

Induction of apoptosis in the neurons of 2–6-week-old mice has been described after infection with herpes simplex virus, Theiler’s murine encephalomyocarditis virus and neurovirulent strains of Sindbis virus [31].

Webb and Rao in their work on 28 human KFD cases with neurological complications described signs of severe headache, neck stiffness, mental disturbance, coarse tremors, giddiness, and severe tremors of arms and legs [32]. Pavri in his clinical study, reported that some of the neurological complications consisted of severe headache, stiff neck, mental disturbance, coarse tremors and abnormal reflexes in some of the KFD-recovered patients [33]. Munivenkatappa and co-workers in their study on human brain autopsies found meningitis, cerebral edema, and inflammatory cells in brain tissue, correlating to the neurological complications and abnormal behavior in the patients [34]. These observations prove neurological complications in human patients who succumb to KFD or suffer with the disease and also in those who recover from the disease. Further studies on KFD-affected human brain lesions may confirm if the pathological findings in mice brain tissue observed in this study simulate with the symptoms and lesions in human brain.

Future studies involving mechanisms leading to apoptosis of neurons during KFDV infection may give newer insights on disease pathogenesis in humans, especially on the neurological symptoms noticed during and after recovering from KFD. The control of virus-induced neuronal apoptosis is likely to be an important determinant of the outcome of encephalitis caused by KFDV and might, in the future, be significant in terms of therapeutic application.
